# Waterborne diseases burden, determinants and health system gaps in Eastern Uganda: a mixed-methods baseline study in the Busoga region

**DOI:** 10.3389/fpubh.2026.1808626

**Published:** 2026-05-25

**Authors:** Dohoon Kim, Jung Sang Hoon, Gabriel Ssekitoleko, Linda Ruvwa, Moses Kyangwa, Damson Atwesigye, Prichard Denzel Kavuma, Brian Ssemwogerere, Sarah Achiro, Ayub Muganda, Benjamin Desmond Tatumwa, Kharim Mwebaza Muluya

**Affiliations:** 1 Korea Foundation for International Healthcare, Kampala, Uganda; 2 Office of the Prime Minister, Kampala, Uganda; 3 Department of Research and Innovations, Busoga Health Forum, Jinja, Uganda; 4 Economic Policy Research Centre, Kampala, Uganda; 5 Department of Public Health, Jinja Regional Referral Hospital, Jinja, Uganda; 6 Department of Public Health, Faculty of Health Sciences, Islamic University in Uganda, Mbale, Uganda

**Keywords:** Busoga region, health system gaps, mixed-methods study, Uganda, water sanitation and hygiene (WASH), waterborne diseases

## Abstract

**Background:**

Waterborne diseases remain a major public health challenge in Eastern Uganda, driven by inequities in water access, sanitation quality, hygiene practice and health system constraints. A baseline study to characterize disease burden, WASH conditions, and care-seeking behaviors in the Busoga sub-region was conducted in Jinja City and Jinja and Iganga Districts.

**Methods:**

Data were triangulated from District Health Information System, household surveys, and Water, Sanitation and Hygiene assessments in schools and health facilities, complemented by observations, focus group discussions, and key informant interviews. Quantitative data were analyzed using STATA, while qualitative data were transcribed and thematically analyzed.

**Results:**

There was higher waterborne disease incidence in Jinja City (5.4%), Jinja District (5.3%), and Iganga (4.6%). However, household surveys confirm 3.8% incidence reported in the program supported villages and 5.9% in the non-program supported villages. Access to improved water sources was highest in Jinja city (72%) and lowest in Iganga (53%), while regular household water treatment remained limited (23%−41%). Although sanitation coverage appeared relatively high (75%−81%), qualitative and school data revealed major quality and equity gaps, including overcrowded school latrines. Handwashing practices were inconsistent, with only 32% of households observed to have soap and water at designated handwashing stations, and only 45% of schools and 46% of health facilities consistently having soap and water available. Waterborne diseases incidence was associated with non-program support villages (RR = 1.72, 95% CI: 1.20–2.45, *p* = 0.003), use of unimproved water sources (RR = 1.85, 95% CI: 1.30–2.65, *p* = 0.001), no treatment of water (RR = 1.78, 95% CI: 1.25–2.55, *p* = 0.002), use of shared sanitation or open defecation (RR = 1.69, 95% CI: 1.15–2.50, *p* = 0.01), lack of a handwashing facility (RR = 2.10, 95% CI: 1.45–3.05, *p* < 0.001) and finally education at secondary level was protective (RR = 0.60, 95% CI: 0.40–0.90, *p* = 0.01). Qualitative findings further highlighted mistrust of piped water quality, cost barriers to soap and water treatment, normalization of childhood diarrhea, overcrowded shared sanitation, and reliance on herbs or drug shops before seeking formal health care.

**Conclusions:**

Waterborne diseases remain high but preventable in Busoga. Strengthening access to safe water, improving sanitation and hygiene infrastructure, and addressing behavioral and health system barriers are critical to reducing disease burden.

## Background

Globally, inadequate access to safe water, sanitation, and hygiene (WASH) remains a major public health challenge, contributing substantially to the burden of infectious diseases. The World Health Organization estimates that unsafe water, sanitation, and hygiene account for a significant proportion of diarrheal diseases, which cause hundreds of thousands of deaths annually, particularly among children in low- and middle-income countries (1, 2, 9). Waterborne infections such as cholera, typhoid, dysentery, and hepatitis A continue to thrive where sanitation infrastructure and hygiene practices remain insufficient. Countries like Bangladesh and Indonesia, initially suffered from poor indicators as low as 50% latrine coverage in early 2000 until community Led Total Sanitation (CLTS) and national sanitation campaigns were introduced and significantly improved these WASH indicators (22). Improving WASH services is therefore recognized as a critical strategy for reducing preventable disease and achieving Sustainable Development Goals related to health and clean water (2, 8).

Across Sub-Saharan Africa, rapid population growth, urbanization, and inadequate infrastructure have intensified challenges in access to safe water and sanitation services. Many urban areas have expanded faster than public health infrastructure, leading to overcrowded informal settlements with limited WASH services and increased exposure to waterborne diseases (2, 3). Flooding, climate variability, and weak waste management systems further contribute to contamination of water sources and recurrent outbreaks of cholera and other enteric infections across the continent. As a result, Africa continues to bear a disproportionate share of global morbidity and mortality associated with inadequate sanitation and hygiene (1–3). Therefore, like in Ghana, it has been suggested that there should be approaches beyond CLTS alone to improve WASH indicators (23).

Uganda's response to sanitation and hygiene challenges is guided by national and international policy frameworks aimed at improving public health and environmental sustainability. These efforts align with Uganda's National Development Plan III and IV, which prioritize improved social service delivery, strengthened resilience, and human capital development as key drivers of national growth (6, 11). In addition, the Ministry of Health's Environmental Health Strategy emphasizes preventive interventions, including improved sanitation, safe water access, and community disease control as critical components of disease prevention (7, 11).

Despite of such guidance, poor sanitation and hygiene are estimated to account for nearly 75% of the country's disease burden, with approximately 23,000 deaths from diarrheal diseases annually, 90% of which are attributed to unsafe water, inadequate sanitation, and poor hygiene practices (7). Although national sanitation coverage has increased modestly to about 81%, challenges such as poor fecal sludge management, limited WASH services in institutions like schools, prisons, and health facilities, and rapid urbanization continue to sustain the transmission of diseases such as cholera, typhoid, dysentery, and other diarrheal infections (7, 20, 24, 25).

In Busoga region, waterborne diseases remain a persistent public health concern, affecting both urban and rural communities. About 10% of outpatients are due to diarrhea, 3% dysentery, and less than one percent for cholera, and typhoid continue to contribute significantly to preventable morbidity and mortality due to inadequate access to safe water, limited sanitation coverage, poor hygiene practices, and gaps in community awareness (18). These infections are strongly associated with exposure to contaminated water sources and poor sanitation systems, particularly in densely populated and low-income settlements where infrastructure and waste management systems are often inadequate (1, 10, 16).

Districts such as Jinja and Iganga include rapidly expanding peri-urban settlements and susceptible flooding communities where drainage systems are weak and sanitation facilities are frequently overwhelmed during heavy rainfall. Seasonal flooding and intermittent water supply often force households to rely on unsafe water sources such as shallow wells, streams, or lake water, increasing the risk of exposure to waterborne pathogens. Similar patterns have been documented across other parts of Eastern Africa where rapid urbanization and limited WASH infrastructure contribute to persistent transmission of diarrheal diseases and enteric infections (2, 3, 17).

Although sanitation coverage in some parts of the region appears relatively high, the functionality, privacy, and safe management of sanitation facilities remain inconsistent. Many households rely on shared or poorly maintained latrines, and access to handwashing facilities with soap and water is limited in homes, schools, and health facilities. These structural constraints, combined with behavioral factors such as normalization of childhood diarrhea, delayed care seeking, and inconsistent water treatment practices, continue to sustain the transmission of waterborne diseases in the region.

To address these gaps, a comprehensive WASH program was initiated in Busoga to strengthen water safety, sanitation access, hygiene behavior changes, and health system readiness. As a first step toward guiding this intervention, a baseline assessment was conducted to characterize the burden of waterborne diseases and identify contextual determinants influencing exposure, prevention, and care-seeking practices in the study area.

## Methods

### Geographical scope of the WASH project

**Figure 1:**
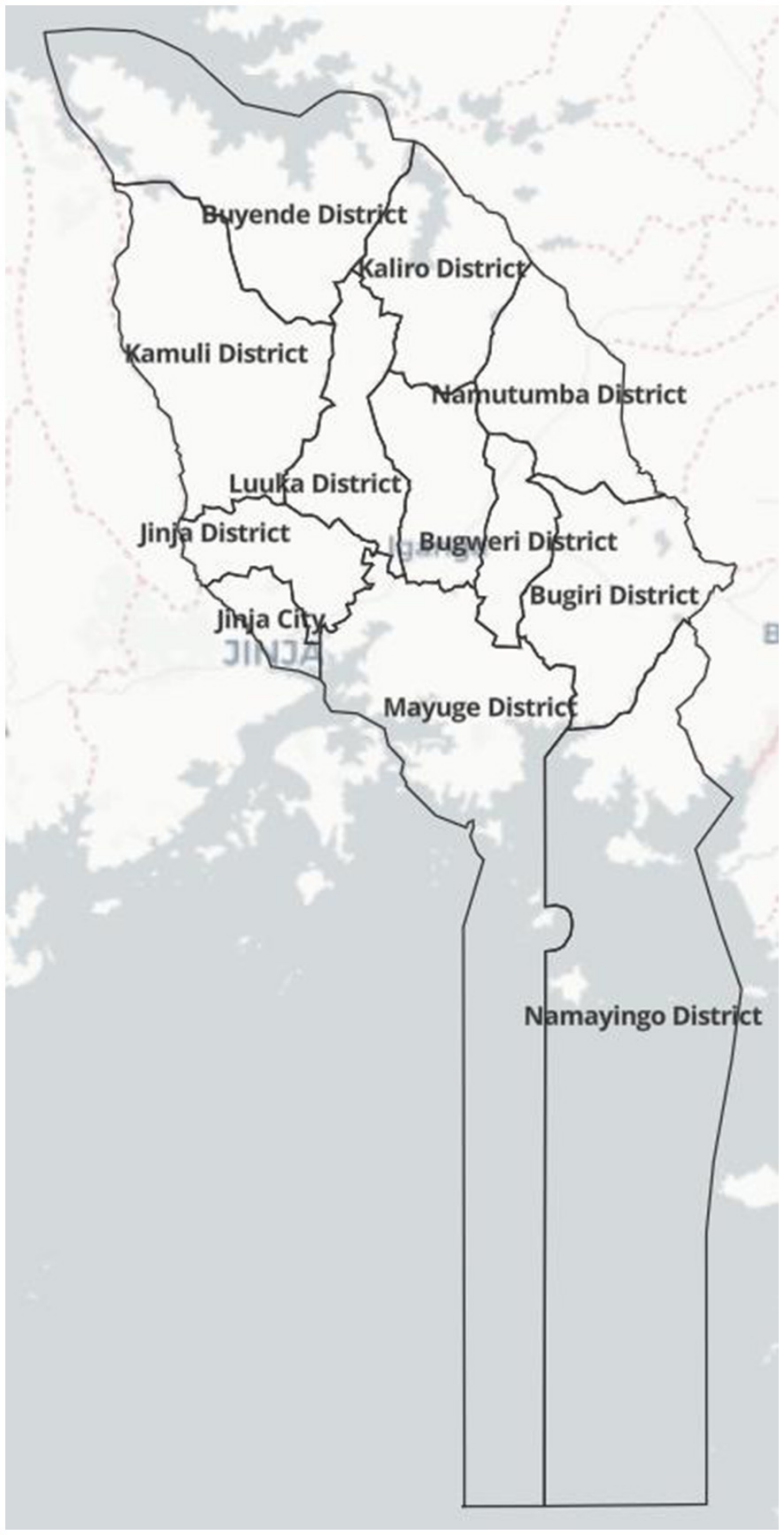
Map of Busoga region indicating Jinja city, Jinja and Iganga districts.

The project is being implemented in Busoga region, in Eastern Uganda and specifically in Iganga District, Jinja District and Jinja City, with a focus on a combination of community, school and health facility settings to address WASH challenges. Busoga region located along the Uganda's busiest Busia/Malaba-Kampala highway with 12 districts (see Figure 1). The Busoga region is located approximately 135 km east of Kampala, the capital of Uganda. The top administrative unit for the region is Jinja city, housing the regional political and administrative headquarters, the Regional Referral hospital and numerous business involving mostly agricultural produces and tourism. The region has a population size of approximately 4.5 million people served by 330 public health facilities and 1,753 primary schools of which 1,132 are government owned and 621 private schools (20).

### Study design and selection of participants

This study used a mixed-methods, quasi-experimental pre-post design using repeated cross-sectional surveys with intervention and comparison clusters in Jinja City, Jinja District, and Iganga District (16, 19). The intervention was implemented in eight villages, two health facilities, and two schools and were purposively selected in collaboration with district health authorities and local government leaders based on three main criteria: (1) documented high burden of waterborne diseases from District Health Information system 2 (DHIS2) and district surveillance reports, (2) evidence of poor WASH indicators such as limited access to safe water, inadequate sanitation facilities, or frequent sanitation-related outbreaks according to the ministry of health annual reports for Busoga region, and (3) feasibility for implementation, while comparison sites were purposively selected from non-program supported villages, facilities, and schools within the same districts, prioritizing nearest-neighbor sites to enhance comparability in socio-demographic and contextual characteristics (19).

For the quantitative component, baseline survey was conducted among households and schools, supplemented with review of DHIS2 data. At baseline, eight program supported villages were purposively selected and eight comparison villages surveyed were also purposively selected, with 280 randomly selected respondents (160 from household members, 50 pupils and 70 health workers) interviewed on administering a structured questionnaire in the intervention group and to the same number in the comparison group (total *n* = 560) by the research assistants. Qualitative data were collected by research assistants through recordings and notetaking in the focus group discussions and key informant interviews from 100 respondents in each group, and structured field observations to explain patterns observed in quantitative findings and to document contextual and implementation factors. The WASH-program has 2 years of intervention starting January 2025, enabling comparison of changes over time between intervention and comparison groups.

### Description of the intervention and the implementation approach

Following the challenges experienced in the study area, a Korean Foundation for International Healthcare (KOFIH) Uganda started the implementation of a WASH program in Iganga District, Jinja District, and Jinja City to strengthen water safety, sanitation access, hygiene behavior changes, and health system readiness, in communities, schools, and health facility settings. The WASH program involves the installation of public handwashing facilities and water purification systems, construction of a community water tank with designated drinking and handwashing points, construction of a modern waterborne toilet and WASH e-content developed by the WASH-program and championed through school health clubs. Across schools, health facilities and entire community, the WASH program emphasizes community engagement, stakeholder collaboration, and integration into existing systems to support sustained WASH practices.

The WASH program has been implemented through community sensitization meetings to raise awareness on safe water use, sanitation, and hygiene practices. School WASH club formation and training to promote hygiene education and the use of WASH e-content to encourage behavior change among pupils. Training of Village Health Teams (VHTs) and health workers to strengthen community engagements, health education, and facility-based WASH practices. In addition, workshops with local leaders and key stakeholders support coordination, community ownership, and integration of WASH interventions into existing local systems.

### Study population and their target areas

The study was conducted in selected communities, schools, and health facilities within Busoga sub region, specifically in Iganga District, Jinja District, and Jinja City. The target population included;

1) Households in highly burdened villages in Nakalama and Bulubandi parishes were selected in the intervention group and Nakabango and Bukaya parishes for the comparison group (see Table 1). Household head or another responsible person in a home responded to the questionnaire administered by the research assistant.
2) School children and teachers in primary schools participated, and were from Mpumudde Estate Primary School and Bunyiiro Muslim Primary School for the intervention group and Kiira primary school and Bunyiiro church of Uganda primary school for the comparison group.
3) District Health Teams, Health workers, heads of facilities at Jinja Regional Referral Hospital, Iganga General Hospital, and lower level health facilities participated in the study. Health workers in Jinja regional referral hospital and Iganga general hospital in the intervention group responded to the administered questionnaire and Iganga municipality and Jinja Central HCIIIs in the comparison group.
4) District leaders, community leaders, VHTs, and local government officials involved in WASH and disease surveillance participated in the study were involved in the FGDs and KIIs.

**Table 1:** Summary showing the study areas and the targeted number of respondents.

District	Sub-county	Parish	Target area	Respondent category	Estimated respondents
Jinja	Budondo	Nakabango (Nakabango A and Butiiki villages)	Community households	Household heads or another responsible person (door-to-door survey)	80
Jinja	Mafubira	Bukaya (Mafubira and Nalambhai villages)	Community households	Household heads or another responsible person (door-to-door survey)	80
Jinja	Mpumudde	Mpumudde (Mpumudde A and B villages)	Mpumudde estate primary school	FGDs with primary school pupils (one FGDs, 10 pupils), and administering of questionnaires to WASH club members	35 (FGDs 10 and administered questionnaires 25)
			Kiira primary school	FGDs with primary school pupils (one FGDs, 10 pupils), and administering of questionnaires to WASH club members	35 (FGDs 10 and administered questionnaires 25)
Jinja	Bugembe	Bugembe central	District and lower local government offices	KIIs with district leaders and FGDs with LC1s	20
Jinja	Jinja Central	Jinja main	Jinja regional referral hospital	Administering of questionnaires to health workers	55
			Jinja central HCIII	Administering of questionnaires to health workers	15
Jinja	Jinja North	Masese	Community centers	FGDs with parents, teachers, health workers (four sessions)	40
Jinja	Jinja Central	Wanyange	Water quality testing facility	KIIs with water quality officials	10
Jinja	Central Division	Civic center	Ministry of LG (via DHO office)	KIIs with administrators linked to Jinja	10
Iganga	Nakalama	Nakalama	Community households	Household heads or another responsible person (door-to-door survey)	80
Iganga	Nakigo	Bulubandi	Community households	Household heads or another responsible person (door-to-door survey)	80
Iganga	Nawanyingi	Bunyiiro	Bunyiiro Muslim Primary School	FGD with primary school pupils (one FGDs, 10 pupils), and administering of questionnaires to WASH club members	35 (FGDs 10 and administered questionnaires 25)
			Bunyiiro C/U Primary School	FGDs with primary school pupils (one FGDs, 10 pupils), and administering of questionnaires to WASH club members	35 (FGDs 10 and administered questionnaires 25)
Iganga	Central Division	Nabidongha	District and lower local government offices	KIIs with district leaders and FGDs with LC1s	20
Iganga	Central Division	Nakavule (Nakavule A and Nkono villages)	Iganga General hospital	Administering of questionnaires to health workers	55
			Iganga municipal council HCIII	Administering of questionnaires to health workers	15
Iganga	Nawanyingi	Magogo	Community centers	FGDs with parents, teachers, health workers (four sessions)	40
Iganga	Northern Division	Kilowa zone	Water quality office	KIIs with water quality officials	10
Iganga	Central Division	Nabidongha	Ministry of LG (via DHO office)	KIIs with administrators linked to Iganga	10

### Sample size determination

Sample size calculations for the Busoga Region WASH study were guided by the minimum detectable effect (MDE) for improved sanitation coverage, which was the primary outcome measured at end line. The assumptions and approach were informed by the WASH for Everyone controlled before-and-after protocol by Chidziwisano K. and others in 2024 and the WASHplus baseline–endline comparison methodology in 2016 (16, 19). A two-tailed test was assumed, with a significance level (α) of 0.05, power of 0.80, and a conservative intra-cluster correlation coefficient (ICC) of 0.10, to account for clustering at village level.

The Busoga WASH study was implemented in three districts in the Busoga Region, Iganga District, Jinja District, and Jinja City, selected due to their high burden of WASH-related diseases and persistently poor WASH indicators extracted from DHIS2 and annual reports to ministry of health. The study adopted a controlled before-and-after cluster design, with both programs supported and non-program supported (comparison) villages included within the study districts (16).

A total of 16 villages were selected, comprising eight program supported villages and eight non-program supported villages, distributed across the three districts. In four selected village, an average of 40 households were sampled using household-level surveys. In two villages, 70 health workers were selected from two health facilities and in two villages 50 pupils from two schools were selected. This resulted to 280 respondents in the program supported villages and 280 respondents in the non-program supported villages, yielding a total sample size of 560 respondents per data collection round, with the same number of respondents targeted at baseline and end line to allow for before-and-after comparisons.

### Sampling techniques

A multi stage sampling approach was used. Districts and city were purposively selected based on documented WASH annual reports and reported waterborne disease burden in the DHIS2. Similarly, sub counties, parishes, and villages were purposively selected using HMIS reports and district surveillance data. Households within selected villages were then sampled randomly to participate in the survey.

Schools were selected purposively to represent different WASH contexts. In schools, respondents were randomly selected from WASH clubs to participate in the WASH program survey. Similarly, health facilities were purposively selected to cover referral hospitals, high volume facilities, and lower level facilities that routinely manage diarrheal and enteric cases, and health workers were randomly selected to participate in the WASH program survey.

For qualitative data, key informants were purposively identified based on their positions, expertise, and direct involvement in WASH-related planning, policy, service delivery, and community governance. The selection ensured representation from multiple levels of leadership and sectors, including health, education, water and sanitation, and local governance. At the district level, senior decision-makers such as Chief Administrative Officers (CAOs) or their deputies, District Health Officers (DHOs), and District Education Officers (DEOs) were selected due to their roles in policy formulation, coordination, and oversight of district programs. Technical personnel, including Environmental Health Officers, Health Inspectors, and Ministry of Health WASH coordinators, were included because of their hands-on involvement in WASH implementation and supervision. At the facility and institutional level, Hospital Directors, Medical Superintendents, and Head Teachers were selected to provide insights into service delivery, institutional practices, and challenges within schools and health facilities. Community-level leadership perspectives were captured through Local Council V (LCV) and Local Council I (LCI) chairpersons, while representatives from the National Water and Sewerage Corporation (NWSC) were included to provide technical perspectives on water supply and quality management.

Participants for focus group discussions were also selected purposively to ensure representation of diverse community perspectives and lived experiences in WASH-related issues. The study targeted key population groups directly affected by WASH conditions, including community members, caregivers, youths, teachers, and school-going children. Within each category, participants were selected based on their residence in the affected areas and their ability to provide relevant experiences and insights. Efforts were made to ensure diversity in age, gender, and social roles to capture a wide range of views and practices related to water, sanitation, hygiene behaviors, and health outcomes.

### Data collection methods and tools

Multiple complementary methods were used to strengthen validity through triangulation. These included;

#### Household surveys

Structured interviewer administered questionnaires were used to collect data on recent episodes of diarrhea, typhoid, cholera, and dysentery, water sources, water treatment practices, sanitation facilities, handwashing behavior, and health seeking patterns. A recall period of up to 5 years was used for reporting episodes of waterborne illness.

#### School surveys and observations

School level tools captured information on pupil exposure to waterborne diseases, school attendance, sanitation infrastructure, handwashing facilities, and availability of safe drinking water. Structured observation checklists were used to verify functionality and use of WASH facilities. Also, structured questionnaires were administered in schools.

#### Health facility data extraction

Standardized data extraction forms were used to review outpatient and inpatient registers, disease specific registers, and DHIS2 or eHMIS reports from 2020 to 2025. Data on diarrhea, cholera, dysentery, and typhoid cases, as well as reporting rates for HMIS 105 and ICCM 097b, were compiled to generate trends over time. Similarly, structured questionnaires were administered to health workers in health facilities.

#### Focus group discussions (FGDs)

FGDs were conducted with caregivers, community members, and schoolchildren to explore perceptions of waterborne diseases, risk factors, water use behavior, sanitation practices, handwashing, and barriers to prevention and care seeking.

#### Key informant interviews (KIIs)

KIIs were held with health facility managers, district health officers, health inspectors, VHT coordinators, district education officers, NWSC managers, CAOs, LCV and LCI chairpersons, ministry of health representatives, and school administrators to gain in depth understanding of health system readiness, surveillance quality, outbreak experiences, and WASH challenges.

#### Direct observation and field notes

Observational checklists were used to document the state and use of latrines, handwashing stations, water points, drainage, and environmental sanitation in communities, schools, and health facilities. Field notes captured contextual issues such as flooding, open defecation, and informal water sources. See Table 2 with a summary of data collection tools and methods.

**Table 2:** Summary of data collection methods and corresponding tools.

Method	Tools used
Household surveys	Structured interviewer-administered questionnaire used. Also, the KoboCollect was uploaded on the smartphones of the data collectors. Other important documents included consent forms, Household listing and pens to the research assistants.
School surveys and observations	Structured interviewer-administered questionnaire used. Also, the KoboCollect was uploaded on the smartphones of the data collectors. WASH infrastructure observation checklist.
Health facility data extraction	Standardized data abstraction form. Outpatient and inpatient registers. Access to DHIS2/HMIS dashboard and some facility registers. Structured interviewer-administered questionnaire also used and uploaded on the smartphone.
Focus group discussions (FGDs)	FGD discussion guide, together with consent forms and audio recorder, notebook for field notes and pens/markers. Flip charts or manila cards were used. Participant attendance sheet were used to register attendance.
Key informant interviews (KIIs)	Semi-structured interview guide was used with consent forms and audio recorder given to the research assistants.
Direct observation and field notes	WASH observation checklist was used. A camera or phone was used to take some photos (with approval/consent).

### Data management and analysis

Quantitative data from household, school, and facility tools were checked for completeness in the field, entered into a secure database (KoboToolBox), cleaned, and analyzed using statistical software STATA *version* 16 (StataCorp LLC. Stata Statistical Software: Release 16. College Station, TX: StataCorp LLC; USA) (21). Descriptive statistics were generated to summarize incidence, prevalence, trends over time, and WASH indicators by district, facility type, and risk group. Time series trends (2020–2025) were produced for key diseases and reporting rates using HMIS and DHIS2 data. Predictors of waterborne diseases were identified using a modified Poisson regression model to estimate adjusted risk ratios with 95% confidence intervals reported at *p* < 0.05.

Qualitative data from FGDs, KIIs, and field notes were audio recorded where consent was granted, transcribed, translated where necessary, and coded using a thematic analysis approach. Emerging themes on risk factors, care seeking, barriers, and system gaps were used to contextualize and explain the quantitative findings.

### Inclusion and exclusion criteria

Participants were included in the baseline assessment if they met clearly defined criteria that ensured they were part of the target population. Household heads 18 and above years old who had resided in the selected communities for at least 12 months prior to the survey were included because of their ability to recall illness episodes and environmental factors and provided informed consent. Within schools, learners and teachers who were present on the day of data collection were included to capture school-level exposure, sanitation behaviors, and school attendance. Health workers and facility managers working in selected health facilities were also eligible, given their critical roles in case management, reporting, and infection prevention. Additionally, community leaders, Village Health Teams (VHTs), and government officials directly involved in WASH coordination, surveillance, or health service delivery were included because of their contextual knowledge and governance roles.

Individuals and entities were excluded from participation when their involvement posed either ethical concerns or risked compromising data accuracy. Visitors and temporary residents who had lived in the area for less than 12 months were excluded due to their limited familiarity with local water, sanitation, and disease patterns. Individuals who were critically ill at the time of data collection and therefore unable to provide informed and voluntary responses were also excluded in line with ethical principles of non-maleficence and respect. In addition, household members, school pupils, or health facilities health workers that declined consent were not included, and those that were repeatedly unavailable after two follow-up visits were also excluded to prevent sampling bias and undue pressure on non-responsive participants.

### Ethical considerations

The WASH Busoga project involved human participants and was conducted in accordance with established ethical standards for research involving human subjects. Ethical principles of respect, beneficence, and confidentiality guided all stages of the assessment. Ethical approval for the WASH program was obtained from Makerere University Institutional Review Board (IRB; MAKSPH-IRB/REC/2025/0448), and administrative clearance was sought from district health and education departments and health facility authorities. Written informed consent was obtained from all adult participants after explaining the purpose of the study, procedures, potential risks and benefits, and their right to withdraw at any time without consequences. For schoolchildren, consent was sought from school authorities and parents where required, and assent was obtained from the pupils themselves. All data were anonymized by removing personal identifiers and assigning unique codes. Completed tools were stored securely, and electronic datasets were password protected. Only the core research team had access to the full data. No individual level findings were shared with third parties, and results are presented in aggregated form.

### Quality assurance

Data collectors were trained on the WASH program protocol, tools, interviewing skills, and research ethics by research team and program managers. Tools were pretested in a non-WASH program community (Bugiri District) with similar characteristics, and refinements were made before full rollout. Daily field supervision, spot checks, and review of completed questionnaires were used to identify and correct errors in real time. Triangulation across household data, facility records, and qualitative evidence was used to improve the robustness and credibility of findings.

## Baseline results

### Socio-demographic characteristics of respondents

Most household members in both groups were male (69.8% in program-supported areas, 74.2% in non-program areas) and married (76.9 vs. 83.5%), with the majority aged above 35 years (51.9 vs. 45.3%), although the non-program group had a slightly higher proportion aged 25–35 years (46.8 vs. 37.1%). A notable difference was observed in education level, where 12.6% of non-program households had no formal education compared to only 2.6% in program-supported areas.

Among health workers, the groups were similar in sex distribution (female 51.8 vs. 50.0%, male 48.2 vs. 50.0%) and cadre, with most being clinicians (80.0 vs. 74.6%) and aged 25–35 years (75.7 vs. 82.4%). Among pupils, females (73.3 vs. 63.7%) and lower-primary learners (80.5 vs. 77.4%) formed the majority in both groups (see Table 3).

**Table 3:** Socio-demographic characteristics of respondents.

Variable	Category	Program supported	Non-program supported
		Frequency (%)	Frequency (%)
*Household members*		*n = 160*	*n = 160*
Sex	Male	112 (69.8)	119 (74.2)
	Female	48 (30.2)	41 (25.8)
Age group	18–24	14 (9)	13 (7.9)
	25–35	59 (37.1)	75 (46.8)
	Above 35	87 (51.9)	72 (45.3)
Education level	No formal education	4 (2.6)	20 (12.6)
	Primary	83 (51.9)	68 (42.2)
	Secondary	64 (40.1)	62 (38.6)
	Tertiary	9 (5.4)	10 (6.6)
Marital status	Single	4 (2.3)	12 (7.3)
	Married	123 (76.9)	134 (83.5)
	Widowed/divorced	33 (20.8)	14 (8.2)
***Health workers***		***n** = **70***	***n** = **70***
Sex	Male	34 (48.2)	35 (50.0)
	Female	36 (51.8)	35 (50.0)
Age	18–24	9 (12.3)	5 (7.6)
	25–35	53 (75.7)	58 (82.4)
	Above 35	8 (12.0)	7 (10.0)
Cadre	Clinician	56 (80)	52 (74.6)
	Non-clinician	14 (20)	18 (25.4)
***Pupils***		***n** = **50***	***n** = **50***
Sex	Male	13 (26.7)	18 (36.3)
	Female	37 (73.3)	32 (63.7)
Class level	Upper primary	10 (19.5)	11 (22.6)
	Lower primary	40 (80.5)	39 (77.4)

### Establishment of baseline indicators of waterborne disease burden, trends, household exposure, and related mortality in Jinja city, Jinja and Iganga districts

#### Incidence of waterborne diseases

Routine DHIS2 data (2020–2023) show that the average incidence rate of waterborne diseases in Busoga is about 4.0%, but the three WASH-program focus areas are clearly above this average, with Iganga District at 4.6% Jinja District at 5.3% and Jinja City at 5.4% (see Table 4). However, household surveys confirm 3.8% incidence reported in the program supported villages and 5.9% in the non-program supported villages (see Table 5). Overall, access to safe water across the three districts ranged from 64% to 79%, while the use of sanitation facilities was generally higher, ranging from 75% to 81.4% as shown in Table 4.

**Table 4:** Overall disease burden (DHIS2 2020–2024) and WASH context in WASH program focus districts.

District/city	Access to safe water (%)	Use of sanitation facilities (%)	Average disease incidence rate (%)	Relative burden in busoga
Iganga district	66	75	4.6	4th highest
Jinja city	79	81.4	5.4	2nd highest
Jinja district	64	81.3	5.3	3rd highest
Busoga overall	56.4	61.3	4.0	Regional average

**Table 5:** Summary table of key indicators—WASH Busoga project (Iganga and Jinja districts and Jinja city).

Objective Area	Indicator	Definition and Unit	Baseline		End line		Overall Project Target 2026	Basis for Target Calculation	Means of Verification	Frequency
			Iganga	Jinja City	Iganga	Jinja City				
Impact: reduced disease burden	Incidence of waterborne diseases	Percentage of reported diarrhea, dysentery, typhoid and cholera cases vs. total population	4.6%	5.4%	≤ 4.5%	≤ 5.3%	Combined incidence ≤ 4.8%	Original 3.5% incidence target revised to 4.8% based on higher baseline (5%) in two focus areas and modest investment, aiming for at least 2% relative reduction.	DHIS2, HMIS 105, facility registers, baseline and end line surveys	Annual, end line
	Mortality attributable to diarrhea and typhoid	Proportion of deaths from waterborne diseases among clinical cases	Not reliably reported (assumed high)	Not reliably reported (assumed high)	≥15% reduction from baseline	≥15% reduction from baseline	At least 15% reduction overall	Target aligns with design matrix goal of 15% mortality reduction through improved IPC, ORS/IV access and earlier care seeking rather than large incidence drops alone.	Facility mortality logs, HMIS, KIIs	End line
Outcome 1: access to safe water	Households with access to improved or safe water	Percent of households using boreholes, protected springs, piped or treated sources	53%	72%	54.06%	73.44%	Average access in both areas ≥63.75%	Design matrix sets 2% increase in overall access (62.5% → 63.75%), allocated proportionally by baseline (Iganga 53% → 54.06%, Jinja 72% → 73.44%) because large gains require large capital not available in this project.	Household survey, water authority records, NWSC data	Baseline, annual, end line
	Households regularly treating drinking water	Percent of households boiling, chlorinating or filtering water	23%	41%	≥30%	≥45%	At least 5–10 percentage point increases overall	Based on behavior-change focus of project and low-cost nature of point-of-use treatment; adopts design matrix range of 5%−10% improvement after awareness and supply support.	Household survey and observation	Mid line, end line
Outcome 2: access and utilization of sanitation	Households using improved sanitation facilities	Percent of households using VIP latrines, flush toilets or septic systems	75%	n.a. in compiled table	≥78%	n.a.	At least 2–3 percentage point increase in each area	Mirrors design matrix assumption that, given fiscal and time limits, the project can realistically drive around 2% improvement in access within 3 km of residence.	Household survey and spot checks	Baseline, end line
	School sanitation adequacy	Pupil to latrine ratio in primary schools	1:135	1:83	≤ 1:80	≤ 1:60	Progressive convergence toward national standard 1:40	Targets chosen as intermediate steps toward national 1:40 standard, reflecting limited but focused school WASH construction budget.	School WASH audit tools, enrolment and facility records	Mid line, end line
Outcome 3: handwashing and hygiene practice	Self-reported handwashing at critical times, households	Percent reporting regular handwashing after toilet and before eating/feeding children	41%	59%	≥48%	≥65%	Overall increase of 5–10 percentage points	Derived from design matrix handwashing target (5%−10% increase from ~47% baseline) using behavior change plus enabling facilities in both areas.	Household survey	Mid line, end line
	Observed handwashing facility with soap and water, households	Percent of households with a functional handwashing station plus soap and water on day of visit	27%	39%	≥35%	≥45%	Overall increase of 8–12 percentage points	Higher gain than self-reported practice assumed because project directly installs/repairs facilities and supports soap/chlorine supply, in line with design matrix 5%−10%+ practice improvement.	Household observation checklist	Baseline, end line
	Schools with functional handwashing stations	Percent of schools with at least one functional handwashing station in daily use	47%	71%	≥60%	≥80%	Overall increase of 10–15 percentage points	Based on output plan to install and rehabilitate school stations; design matrix anticipates ~10%−15% gain across institutions with new hardware and WASH clubs.	School WASH audit	Annual, end line
	Schools with soap consistently available	Percent of schools where soap is present and replenished at handwashing points	38%	55%	≥50%	≥65%	Overall increase of 10–15 percentage points	Follows design matrix assumption that regular budgeting and supervision can lift soap availability by ~10%−15% over 2 years.	School audit, interviews with teachers	Mid line, end line
	Health facilities with functional handwashing stations	Percent of health facilities with functional handwashing stations at key points of care	38%	68%	≥50%	≥78%	Overall increase of 10–15 percentage points	Drawn from design matrix: facility WASH upgrades and IPC training expected to yield 10%−15% increase in functionality.	Facility WASH audit	Annual, end line
	Health facilities with soap and water consistently available	Percent of health facilities with reliable soap and water at handwashing points	35%	61%	≥48%	≥70%	Overall increase of 10–15 percentage points	Target mirrors matrix expectation of improved supply chains and budgeting for soap/water, giving 10%−15% gain over baseline.	Facility audit, stock and utility records	End line
Outcome 4: awareness and behavior change	Knowledge of at least two waterborne diseases	Percent of respondents who can correctly name ≥2 waterborne diseases	87% (combined)	87% (combined)	≥92%	≥92%	Composite awareness 82–90% by 2026	Based on design matrix aim of 10%−20% relative awareness improvement from composite 74.67%, with ceiling effect because baseline already high for disease naming.	Pre and posttests, household survey	Per campaign, mid line, end line
	Knowledge of at least two preventive actions	Percent of respondents naming ≥2 preventive actions	76% (combined)	76% (combined)	≥85%	≥85%	As above	Uses same 10%−20% improvement band in design matrix, focusing on preventive actions where baseline lower than disease naming.	Surveys, school quizzes	Per campaign and end line
	Correct preparation of ORS	Percent able to correctly describe/demonstrate ORS preparation	61% (combined)	61% (combined)	≥75%	≥75%	As above	Higher growth margin selected because baseline is lowest of the three awareness indicators and strongly linked to practical demonstrations in training plan.	Practical demonstration checklist	Mid line, end line
Outputs: systems and collaboration	Number of signed MoUs	MoUs with key government and institutional partners	0	0	≥2 Iganga MoUs	≥2 Jinja City MoUs	Total ≥4 MoUs	From design matrix target: three MoUs in 2025 and one in 2026 to institutionalize collaboration in both areas.	Signed agreements	Annual, end line
	Completed water purification or safe water facilities	Number of purification units, tanks or filters in schools, communities, facilities	0	0	≥4 facilities	≥4 facilities	Total ≥8 facilities	Directly reflects project plan: one tank + three filters in 2025 and four filters in 2026 across high-density public sites.	Completion reports, photos, commissioning min	On completion, end line
	Completed sanitation facilities	Number of toilets and handwashing stations in schools, communities, facilities	0	0	≥4 facilities	≥4 facilities	Total ≥8 sanitation facilities	Mirrors design matrix (one toilet + three stations in 2025; four stations in 2026), roughly half allocated per area.	Engineering reports, photos, handover forms	On completion, end line
	Awareness campaigns and participants reached	Number of campaigns and people reached through SBCC/WASH awareness	0 campaigns, 0 participants	0 campaigns, 0 participants	≥3 campaigns and ≥150 participants	≥4 campaigns and ≥200 participants	Total ≥7 campaigns and ≥350 participants	From project plan: three campaigns/150 people in 2025 plus four campaigns/200 people in 2026 (minimum 50–100 per event).	Attendance sheets, activity reports	Annual, end line
	Satisfaction with awareness campaigns and e-content	Percent of participants expressing satisfaction with campaigns/materials	0%	0%	≥75% (by 2025)	≥80% (by 2026)	75%−80% overall	Uses design matrix satisfaction band of 75%−80%, with incremental improvement across years as content is refined.	Post-activity survey tools	After each activity, annual summary
	Capacity building courses and participants	Number of structured training courses and participants	0 courses, 0 participants	0 courses, 0 participants	Contribute to 160 total participants	Contribute to 160 total participants	≥4 courses and ≥160 participants	Directly from matrix: four courses in 2026 with at least 40 participants each, shared across Iganga and Jinja City.	Training reports, pre and post tests	End of 2026
	Promotional videos and media coverage	Number of videos and positive media pieces on the project	0 videos, 0 articles	0 videos, 0 articles	≥1 video and ≥2 media pieces with Iganga content	≥1 video and ≥2 media pieces with Jinja City content	≥2 videos and ≥4 media pieces	Based on planned production of one video per year and at least two media publications per year as per design matrix.	Media tracking logs, links, copies	Annual

**Figure 2:**
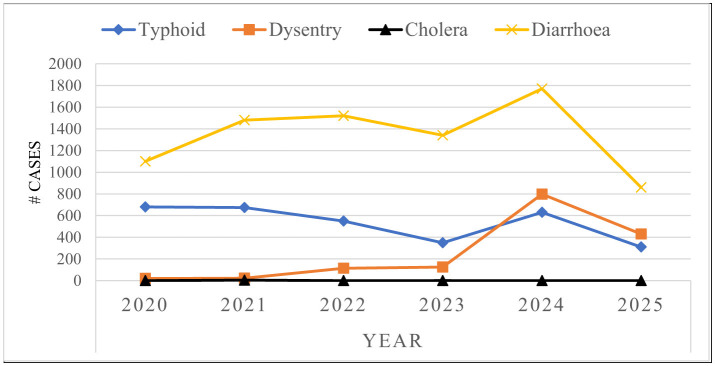
Waterborne diseases trend from 2020 to 2025 in the three districts of WASH program implementation.

The consolidated data shows that diarrhea remains the most prevalent waterborne disease across all three districts, with consistently high case numbers every year. Dysentery cases rose sharply in 2024, especially in Iganga and Jinja District, indicating worsening sanitation challenges. Typhoid displays fluctuating trend in the 5 years, while cholera appears episodic, occurring mainly during times of floods. Overall, the trends reflect persistent vulnerability to WASH-related illnesses, particularly in peri-urban and rural settings (see Figure 2).

Qualitative data strongly reinforce this picture in the program supported and non-program supported villages. In FGDs, participants from peri-urban areas such as Mpumudde, Nakavule, Kyamagwa, Masese and Walukuba repeatedly described frequent episodes of “stomach problems” and unexplained fevers, especially among children. One respondent from Mpumudde explained;

“*Most people here fetch water from the lake or from shared taps which are not always reliable. When the tap is off, we go to the lake, and that's when the children fall sick.”* [KII 23 in the non-program supported villages]

Others in Iganga reported that diarrhea is so common that it is often seen as “normal” for children, which delays timely care seeking.

During the key informant interviews, it profoundly came out that the high disease burden was strongly linked to gaps in safe water access and inconsistent sanitation use, especially in Iganga where water access remains low. In contrast, in Jinja City and Jinja District, where sanitation coverage is higher, disease incidence remains among the highest, due to infrastructure without consistent hygiene practices.

**Figure 3:**
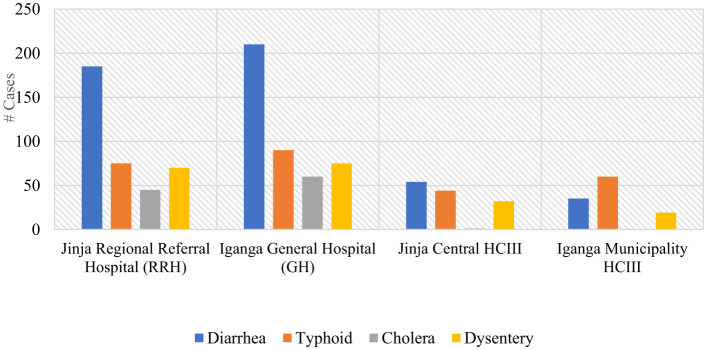
Waterborne diseases in program supported Iganga General Hospital and Jinja Regional Referral Hospital and program non-supported Jinja Central and Iganga Municipality HCIIIs.

Important to note, is that in the WASH-program supported facilities, severe waterborne disease cases remain consistently higher especially in Iganga Hospital than in Jinja RRRH. Jinja Central and Iganga Municipality HCIIIs in the non-supported facilities have registered low cases of waterborne diseases according to DHIS2 data 2020–2024 (see Figure 3).

Taken together, the quantitative and qualitative evidence confirm that the project begins in a context of elevated disease incidence, with hotspots in peri-urban and susceptible to flooding areas. The revised impact target of reducing incidence from around 5%−4.8% or below by 2026 is therefore ambitious but appropriate, given the scale of investment and geographic focus.

#### Trends, household exposure and risk factors

Based on Figure 2, across 2020–2024, facility records show a rising trend in diarrhea, typhoid and dysentery cases up to 2024, with a modest decline in 2024. Cholera appears in episodic spikes, particularly in Iganga General Hospital located in Iganga and Jinja regional referral hospital and Jinja central HCIII in Jinja during 2021–2023. This is consistent with community narratives. Respondents in Iganga and Jinja described a pattern where water sources change with seasons and supply interruptions, often forcing them to shift from taps to lakes, swamps or shallow wells, especially when piped water is off. It was the same narrative in the program supported villages and non-program supported villages.

Qualitative data highlight three key risk amplifiers:

1) Unsafe water sources and low treatment: many households defined safe water correctly “boiled,” “clean from taps,” “water that does not cause illness” but admitted that they rarely boil or chlorinate water due to cost or time. [KII 03 in the program supported villages]
2) Overcrowded, shared sanitation: respondents reported sharing latrines with five or more households, creating unsanitary conditions; some admitted to occasional open defecation when latrines were full or far. [KII5 and 6 in the non-program supported villages]
3) Delayed care seeking: several FGDs revealed a reliance on herbs and self-medication. One mother from Iganga noted, “*When our children get stomach problems, we usually give them herbs or buy cheap medicine, but many times it gets worse*.” [Male participant in the non-program supported villages]

These qualitative insights align with the higher household reporting of illness and explain why peri-urban and low-lying settlements are repeatedly identified as hotspots.

#### Mortality related to diarrhea, cholera, dysentery and typhoid

While precise mortality rates could not be calculated from available records, KIIs with health facility managers and district teams consistently highlighted diarrhea and typhoid as leading causes of under-five deaths, particularly during rainy seasons and during the 2024 Aeromonas-related outbreak. Jinja Regional Referral Hospital noted that many children arrive with severe dehydration after prolonged diarrhea and vomiting, emphasizing that

“… *Some of these cases could be prevented if clean water and sanitation were accessible.”* [KII 11and respondent from the WASH-program supported facility]

Additional qualitative evidence indicates:

1) *Health workers estimate that around 20 percent of diarrhea cases at JRRH arrive with complications, and 3–5 percent of these complicated cases result in death, often due to late referral and lack of early ORS use at home*. [KII 3 from the WASH-program supported facility]
2) *Health inspectors acknowledged that deaths at home or in transit are rarely captured, which explains why the actual mortality burden is higher than reported*. [KII 33 from the program supported villages]
3) *Communities in susceptible to flooding areas described episodes where “many people in the same village” developed watery diarrhea during rainy periods, and some “died before reaching the hospital,” especially where roads become impassable*. [FGD in the non-program supported villages]

### Assess access to, utilization and functionality of WASH services at household, school, and health-facility levels in the study area

#### Access to and use of clean and safe water

Quantitative data show that between 2020 and 2024, household access to improved water sources was slightly different in the three districts (Jinja City, Jinja District and Iganga District). Jinja City had 72% access to clean water in the program supported villages and 69% in the non-program supported villages, Jinja District had 56 and 60%, respectively, and Iganga District had 53 and 56%, respectively.

However, household treatment and safe utilization lag behind. Only 23%−41% of households across the three areas reported regularly boiling or chlorinating water. For the program supported and non-program supported villages (see Table 6). This was echoed by qualitative findings:

*Many caregivers said they know boiling is important but “do not have enough firewood or charcoal” or feel that “tap water looks clean, so there is no need.”* [FGD in the program supported villages]

**Table 6:** Household water access and treatment, 2024 baseline.

District/city	Clean water access (%)		Households treating water (%)		Qualitative challenges identified
	PSV	N-PSV	PSV	N-PSV	
Jinja city	72	69	41	40	Intermittent supply, mistrust of piped water when it appears “muddy”
Jinja district	56	60	29	28	Broken boreholes, long distances; some families resort to streams in dry spells
Iganga district	53	56	23	25	Reliance on shallow wells; cultural belief that “clear” water does not need boiling.

PSV, program supported villages; N-PSV, non-program supported.

FGDs and KIIs also revealed mistrust of water quality in some piped systems; households reported that “sometimes the water comes out brown or with particles,” which pushes them back to unsafe surface sources. These perceptions help explain the gap between access and effective utilization, and they justify the project's focus on both hardware upgrades and behavior change. The outcome target of a 2% increase in access in the project area remains realistic, but impact on disease will depend on addressing these utilization barriers highlighted in community narratives.

#### Access to and use of sanitation facilities

Regionally, sanitation facility utilization averages around 61.3%, with Iganga at 75%, Jinja city at 81.4% and Jinja District at 81.3%. However, detailed school and community data show that these figures mask serious quality and equity challenges. For example, school latrine-to-pupil ratios in all the groups are 1:83 in Jinja City and 1:135 in Iganga, far above national standards of 1:40. In FGDs, girls reported skipping school during menstruation due to lack of privacy, water and menstrual hygiene materials.

“*When my period comes, I just stay home. The toilets at school have no water, no soap and everyone can see you enter. You feel embarrassed.”* [A 14-year-old girl said during an FGD at Mpumudde Estate Primary School]

Community members in the WASH-program supported villages in susceptible to flooding parishes described collapsing pits and flooded latrines, which drive periodic return to open defecation.

“*When it floods, the latrines collapse and we cannot use them. We go back to the bush or the swamp because there is nowhere else to go.”* [Explained a community member from Masese during a village discussion]

Participants frequently cited cost (for slabs and pit emptying), distance and lack of privacy as reasons for not consistently using the available facilities. These qualitative insights support the project's decision to go beyond simple coverage and track actual utilization and user experience.

#### Health facility and WASH facilities utilization

Quantitative data comparison of the program supported and non-program supported villages show that a high proportion of households live within 3 km of a health facility (91 and 90%, respectively) in Jinja City, 67 and 58% in Jinja District and 61 and 60% in Iganga (see Table 7).

**Table 7:** Health facility access vs. utilization (2024 baseline).

District	Households within 3 km		Utilization level		Key challenges identified
	PSV	N-PSV	PSV	N-PSV	
Jinja city	91%	90%	Moderate-high	Moderate-high	Overcrowding, poor sanitation, mistrust of care.
Jinja district	67%	58%	Moderate-low	Low	Long distances, understaffed facilities, reliance on home remedies
Iganga district	61%	60%	Low	Low	Poor WASH in facilities, water shortages, limited drugs

PSV, program supported villages; N-PSV, non-program supported villages.

However, FGDs and KIIs reveal why this does not always translate into timely and effective care.

1) *Caregivers described long waiting times, stockouts of medicines and dirty or blocked toilets at some health centers, which discourage non-emergency visits*. [FGD in a facility in the program supported villages]
2) *Health workers acknowledged periods where water is not available at the facility, forcing staff and patients to fetch water from neighboring compounds or pay for jerrycans*. [KII 24 and KII 29 in a facility in the program supported villages]
3) *Several community members, especially men, reported a preference for buying drugs from private clinics or drug shops, or using herbs, before considering formal facilities*. [KII 05 in the non-program supported villages]

**Figure 4:**
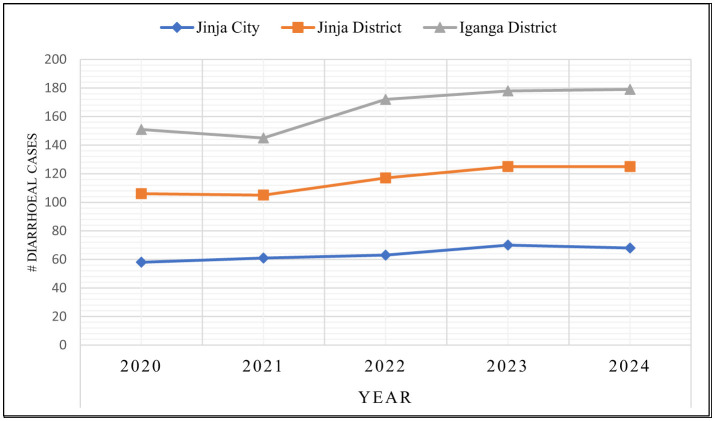
Trends of outpatient attendance due to diarrhea in the three districts.

Consider the trends of ooutpatient attendance for diarrhea from 2020 to 2024, increased steadily across the three districts (see Figure 4), though suggesting gradual improvements in health-seeking behavior, but rural areas still lag behind.

Even where boreholes exist, KIIs with local leaders and water committees revealed many factors contributing the waterborne disease burden. For example; breakdowns can take weeks to months to repair, leading to queues, conflict and reversion to unsafe sources. Taken together, the quantitative and qualitative data show that the project's outcome objective of improving facility utilization by at least 15 percent will require simultaneous action on; functionality and maintenance; perceived and actual quality of services; and community trust and feedback mechanisms.

### WASH awareness, participation in WASH sensitization activities, and handwashing practices

#### Participation and satisfaction in awareness and training activities

Between March 2025 and July 2025, the WASH-program and partners reached 673 participants through data collection, community sensitization meetings, school health club activities, VHTs and health worker trainings and local leader workshops. This represents 91 percent of the target 740 individuals for structured capacity building by 2026, showing strong early momentum.

Qualitative feedback from participants highlights both enthusiasm and clear learning gains. In Busedde Subcounty, one mother reflected;

“*I used to think diarrhea is normal for children. But after the training, I understood it can kill and how to prevent it.”* [A parent in the FGD in the non-program supported villages]

The early phase of capacity building demonstrated strong community interest and a clear gap in prior knowledge. In many sessions, participants struggled to name more than one waterborne disease at the beginning. However, post-session feedback and assessments showed substantial knowledge gains and satisfaction with delivery approach, especially when practical demonstrations were included like the tippy tap construction, ORS preparation, water chlorination, amongst others.

**Figure 5:**
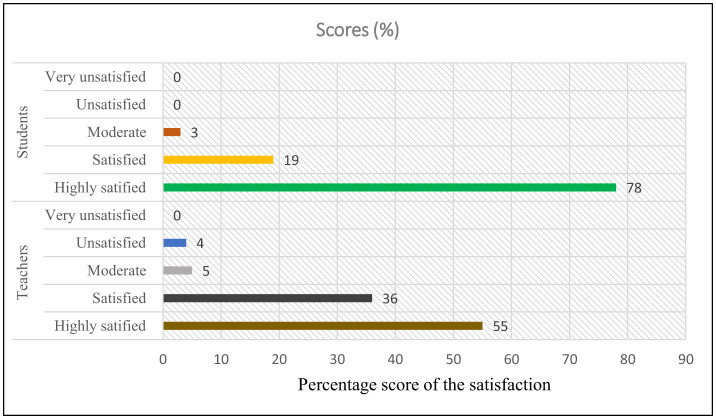
Overall e-content satisfaction by pupils and teachers during pre-testing.

For example, in the program supported schools, the e-content, WASH debate and the music, dance and drama awareness campaigns, was appreciated by teachers and pupils, describing them as “easy to remember” and “better than just talking.” At the same time, some rural participants requested shorter sessions and more use of local languages, noting that long, English-heavy meetings were tiring and hard to follow. Post-session assessments show that 78% of participants expressed overall satisfaction, 81% found the content relevant and practical and 77% rated delivery methods (drama, role plays, demonstrations) as effective. See Figure 5 as an example of the satisfaction assessment for the e-content pre-testing which was presented to teachers and pupils of Bunyiiro Muslim primary school and Mpumudde Estate primary school in the program supported schools.

These numbers, supported by rich qualitative testimonies, indicate that the project is on track to meet output targets of 75%−80% satisfaction with campaigns and materials, while pointing clearly to areas where adaptation is needed (session length, language choice and male engagement).

“*The lessons were exciting and easy to follow. The videos helped us understand faster, and I felt more confident using the WASH practices at home.”* [Class leaders said in one of the FGD with the pupils in Mpumudde Estate Primary School, reflecting the high satisfaction with campaign materials]

“*The sessions are good, but sometimes the sessions are too long, and some pupils who speak little English may get lost. If we involve more use local language, the message will reach many more families.”* [a teacher from Bunyiiro Muslim Primary School mention during KII (KII 31)]

#### Awareness levels, behavior change and gaps

**Figure 6:**
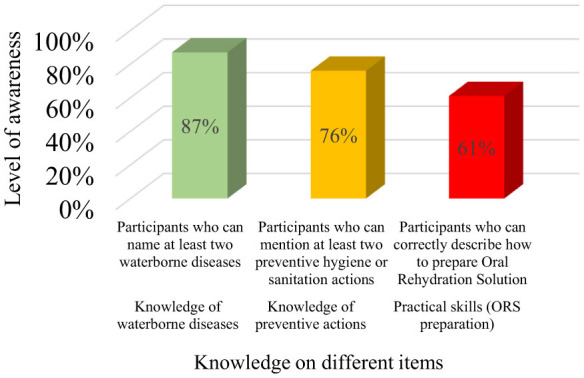
Level of awareness on waterborne diseases, preventive actions and ORS preparations.

Quantitatively, awareness levels are already moderately high. That is, 87 percent of participants can name at least two waterborne diseases, 76 percent can mention at least two preventive actions and 61 percent can correctly describe how to prepare ORS in all the categorized villages. See Figure 6.

This is slightly consistent with DHIS2 trends, which show that the proportion of people in Busoga who can name at least two preventive actions increased from 58% in 2020 to 72% in 2024, though still below national averages.

Qualitative data add nuance. For example; Many participants know disease names and basic prevention, but still perceive diarrhea as a “normal” childhood event that does not require urgent attention.

“*We all know diarrhea is common in children. It comes and goes. Unless the child becomes very weak, we don't rush to the hospital.”* [A mother during a FGD in Bulubandi Central A, Iganga District a program supported village stated]

In several FGDs, caregivers admitted that they do not always put knowledge into practice because of cost (soap, fuel, chlorine), time constraints or competing priorities.

“*They tell us to boil water, to buy chlorine, to keep soap at the handwashing station, but honestly, sometimes you choose food first.”* [A mother in Mpumudde village a program supported in Jinja City also mentioned during FGD]

Schoolchildren could often repeat key messages but pointed out that handwashing stations lacked soap and that water at school was “sometimes dirty” or not available, making it difficult to act on what they had learned.

One P7 pupil in Iganga in Bunyiiro Muslim primary school in a program supported school commented in FGD. “*We sometimes get water from the borehole near the fence, but it's not clean. My friend had typhoid.”*

#### Handwashing practice rates and barriers

Quantitative indicators show a growing but still inadequate level of handwashing practice. For example, at household level, 49% self-report regular handwashing at critical times, but only 32% had both soap and water at a designated handwashing station on the day of observation (39% in Jinja City, 31% in Jinja District, 26% in Iganga). At school level, 57% of schools have functional handwashing stations, but only 45% have soap consistently available. Lastly, at health facilities, 52% have functional stations and 46% have soap and water consistently available, with better performance in Jinja City than in rural Iganga and Jinja District. In comparison with the PSV and the n-PSV (see Table 8).

**Table 8:** Handwashing practice indicators across households, schools, and health facilities (baseline, 2025).

Level of Analysis	Indicator	Overall (%)	Jinja city (%)		Jinja district (%)		Iganga district (%)	
			*PSV*	*N-PSV*	*PSV*	*N-PSV*	*PSV*	*N-PSV*
Households	Self-reported regular handwashing at critical times	49	59	57	46	47	41	42
	Observed handwashing facility with soap and water	32	39	40	31	30	26	28
Schools	Schools with functional handwashing stations	57	71	73	52	51	47	46
	Schools with soap consistently available	45	55	55	41	43	38	36
Health facilities	Facilities with functional handwashing stations	52	68	65	49	52	38	39
	Facilities with soap and water consistently available	46	61	60	42	40	35	37

Table 8 clearly shows that Iganga District consistently performs lowest across all indicators, with the smallest proportion of households, schools, and health facilities having functional handwashing stations or soap and water available.

From a qualitative perspective, households frequently mentioned prioritizing soap for laundry and bathing, rather than handwashing, especially when finances are tight. Some respondents admitted that they “just rinse with water” after latrine use, believing it is good enough. Health workers in rural facilities reported that interrupted water supply and irregular soap procurement make it difficult to enforce infection prevention standards.

“*When money is little, soap is for washing clothes and bathing. We cannot waste it on washing hands every time.”* [A mother, and respondent in Bulubandi Central A in Iganga District said during FGD]

“*After using the toilet, I just rinse with water. It is enough… we have done that since childhood.”* [A male respondent and a parent in Jinja District mentioned during FGD]

“*Sometimes we tell patients and staff to wash hands, but there is no soap or water in the tank. How do we enforce hygiene like that?”* [A Health Worker in Iganga General Hospital lamented during KII (KII 09)]

National DHIS2 data show that Busoga's household handwashing rate with soap and water improved from 29 percent in 2020 to 44 percent in 2024, an increase of 15 percentage points, but still 4–5 points below national averages.

“*The project's target of increasing handwashing practice by 5–10 percent (to roughly 49–52 percent by 2026) is therefore realistic, but will depend heavily on making soap and low-cost handwashing infrastructure (for example, tippy taps) more available, embedding handwashing into daily routines at schools and health facilities, and keeping behavior change communication visible and practical, using local champions and radios, and social media*” according to the a KII 14 and KII 21 from health department of Iganga District.

### Determinants of the incidence of waterborne diseases in Busoga region

See Table 9 showing determinant variables at baseline level. Households in non-program support villages had a higher risk of waterborne diseases compared to program support villages (RR = 1.72, 95% CI: 1.20–2.45, *p* = 0.003). Education at secondary level was significantly protective against waterborne diseases (RR = 0.60, 95% CI: 0.40–0.90, *p*= 0.01). Use of unimproved water sources was significantly associated with higher risk (RR = 1.85, 95% CI: 1.30–2.65, *p* = 0.001). Similarly, households that did not treat water had increased risk (RR = 1.78, 95% CI: 1.25–2.55, *p* = 0.002). Use of shared sanitation or open defecation was associated with higher risk compared to private latrine use (RR = 1.69, 95% CI: 1.15–2.50, *p* = 0.01), and lack of a handwashing facility was strongly associated with increased risk (RR = 2.10, 95% CI: 1.45–3.05, *p* < 0.001).

**Table 9:** Determinants associated with incidence of waterborne diseases in program supported and non-program supported villages.

Variable	Category	PSV incidence *n*/*N* (%)	N-PSV incidence *n*/*N* (%)	Crude RR (95% CI)^*^	Adjusted RR (95% CI)^*^
Program exposure	PSV	6/160 (3.8)		1.0 (Ref)	1.0 (Ref)
	N-PSV		9/160 (5.9)	1.65 (1.15–2.35)^**^	1.72 (1.20–2.45)^**^
Sex of household head	Male	7/112 (6.3)	8/119 (6.7)	1.0 (Ref)	1.0 (Ref)
	Female	4/48 (8.3)	6/41 (7.3)	0.94 (0.68–1.30)	0.92 (0.651–0.30)
Age group	18–24	2/14 (14.3)	2/13 (15.4)	1.0 (Ref)	1.0 (Ref)
	25–35	4/59 (6.8)	5/75 (6.7)	0.90 (0.62–1.32)	0.88 (0.60–1.30)
	>35	5/87 (5.7)	7/72 (9.7)	0.75 (0.521–0.08)	0.72 (0.50–1.05)
Education level	No formal education	1/4 (25.0)	4/20 (20.0)	1.0 (Ref)	1.0 (Ref)
	Primary	5/83 (6.0)	6/68 (8.8)	0.78 (0.55–1.12)	0.76 (0.52–1.12)
	Secondary	5/64 (7.8)	4/62 (6.5)	0.62 (0.42–0.92)^*^	0.60 (0.40–0.90)^*^
	Tertiary	0/9 (0.0)	0/10 (0.0)		
Water source	Improved	4/110 (3.6)	5/120 (4.2)	1.0 (Ref)	1.0 (Ref)
	Unimproved	7/50 (14.0)	9/40 (22.5)	2.00 (1.45–2.75)^***^	1.85 (1.30–2.65)^**^
Water treatment	Yes	3/60 (5.0)	3/63 (4.6)	1.0 (Ref)	1.0 (Ref)
	No	8/100 (8.0)	11/97 (11.6)	1.90 (1.35–2.65)^***^	1.78 (1.25–2.55)^**^
Sanitation use	Private latrine	4/89 (4.4)	5/95 (5.3)	1.0 (Ref)	1.0 (Ref)
	Shared/open defecation	7/71 (10.0)	9/65 (13.8)	1.85 (1.30–2.65)^**^	1.69 (1.15–2.50)^*^
Handwashing facility	Soap and water available	3/70 (4.3)	4/75 (5.3)	1.0 (Ref)	1.0 (Ref)
	Not available	8/90 (8.9)	10/85 (11.8)	2.30 (1.65–3.20)^***^	2.10 (1.45–3.05)^***^
Distance to health facility	≤ 3 km	6/109 (5.5)	7/101 (7.0)	1.0 (Ref)	1.0 (Ref)
	>3 km	5/51 (10.0)	7/59 (11.7)	1.35 (0.98–1.88)	1.32 (0.95–1.85)

^*^p < 0.05, ^**^p < 0.01, and ^***^p < 0.001.

Ref, reference; RR, risk ratio; CI, confidence interval; PSV, program support village; N-PSV, non-program support village.

n/N is the number of respondents who had episodes of at least three waterborne diseases (diarrhea, typhoid, dysentery or cholera) out of the total number of study respondents.

### Examining the adequacy of WASH infrastructure, delivery systems, educational content and publicity mechanisms

At baseline, most infrastructure, systems, content and publicity indicators are formally at zero, as the project is concluding its preparatory and early implementation phase. However, both quantitative records and qualitative observations show that foundational outputs are already emerging.

Looking at infrastructure, community members and schoolchildren in Nakalama Town Board, Bulubandi Central A, Bunyiiro Muslim Primary School and Mpumudde Estate Primary School reported noticeable improvements where handwashing facilities, water purification systems and a new toilet have started to be installed. Caregivers at Iganga General Hospital also acknowledged that new WASH infrastructure is being constructed at the main entrance and it will make it easier to keep clean, although consistent supplies (water, soap, chlorine) remain essential.

“*The new handwashing stations at the entrance will help us keep clean, but they must always have water, soap, even chlorine. Otherwise, people will just pass by without using them.”* [Caregiver, Iganga General Hospital mentioned during FGD]

On collaboration and systems, KIIs with district officials, hospital managers and school administrators confirm strong willingness to collaborate and an emerging sense of shared ownership. Several stakeholders described the WASH-program as timely and as a chance to the district.

“*This project has come at the right time. We already began work with CLTS and school health clubs, and now we finally have a partner to help us strengthen what we started. If we keep working together, we can institutionalize these WASH actions, not just do temporary activities.”* [KII 08 at Jinja District Health Office]

Lastly, about publicity, at baseline, WASH-program publicity was largely introductory and awareness-oriented. Communication focused on sensitizing district leadership, health managers, school administrators, and community stakeholders about the project's goals, scope, and expected benefits. Initial visibility was achieved through stakeholder meetings, district launch briefings, and the circulation of concept notes and introductory materials. These early engagements were complemented by community dialogues and school-level orientations, which created foundational understanding and curiosity about the WASH interventions. While the reach was modest, the messaging succeeded in positioning the project as a credible initiative backed by institutional partners.

Early results started with the program launch and immediate installation of handwashing facilities, school sanitation improvements, and demonstration sessions which drew public interest, and further amplified by local leaders and frontline workers who shared experiences in their networks. Early project visibility was strengthened through school health clubs, community sensitization meetings, and health worker trainings, which became both information channels and advocacy platforms. These engagements helped transform initial awareness into a stronger sense of ownership, reflected in positive coverage from stakeholders and increased demand for participation in subsequent project phases.

In summary, the baseline confirms that the project's impact, outcome and output targets are grounded in the current epidemiological and WASH situation, and that both quantitative trends and qualitative experiences point to the same conclusion; waterborne disease burden in Jinja city, Jinja and Iganga districts is high but modifiable. With the planned combination of infrastructure, behavior change, capacity building and stronger local systems, the log frame targets are challenging yet feasible, provided implementation stays on schedule and responsive to the lived realities captured in the qualitative data.

## Discussion

The baseline evidence indicates that the burden of waterborne diseases in both project-supported and comparison group remains substantially higher than the Busoga sub-regional average, driven primarily by diarrhea, dysentery, and typhoid. Similar spatial inequalities in waterborne disease burden have been documented across eastern Uganda, where peri-urban and densely populated districts consistently report higher diarrheal morbidity due to unsafe water sources, poor sanitation coverage, and weak fecal sludge management systems (1, 2). National surveillance data show that diarrheal diseases remain among the top causes of outpatient attendance in Uganda, with higher incidence observed in urbanizing settings such as Jinja City and Iganga District, where rapid population growth has outpaced WASH infrastructure development (3). Despite these gaps, improvements in VHT/ICCM reporting between 2020 and 2024, for example Iganga increasing from 73 percent to 95 percent and Jinja District from 81.8 percent to 96.2 percent, provide a stronger platform for monitoring disease and mortality trends over the project period and support the program target of a 15 percent reduction in deaths from the four key waterborne diseases.

Household-level survey findings showing that 30%−34% of households reported at least one episode of diarrhea, cholera, dysentery, or typhoid in the previous year are consistent with prior evidence demonstrating that community-reported morbidity substantially exceeds health facility–based incidence estimates. Multiple studies in Uganda and similar low-income settings have shown that only a fraction of diarrheal episodes, particularly among children, are reported to formal health facilities, due to self-treatment, delayed care seeking, or normalization of symptoms (4–6, 20). Household surveys in the present study further confirm that approximately 30%−34% of households experienced at least one episode of diarrhea, cholera, dysentery, or typhoid in the previous year, underscoring a substantial hidden burden that goes beyond what routine incidence rates alone capture across the study groups. As a result, routine facility data often underestimate the true burden of WASH-related diseases, especially in communities where access barriers and informal care pathways dominate.

Qualitative accounts from peri-urban communities further reinforce this pattern. Participants' descriptions of frequent stomach illnesses, seasonal disease peaks, and the normalization of diarrhea among children align with anthropological and public health studies documenting how repeated exposure to enteric infections leads to social normalization of illness (12). When diarrhea is perceived as a common or unavoidable part of childhood, caregivers are less likely to seek timely care or adopt preventive behaviors, even when basic knowledge of hygiene and disease transmission is present (14). This normalization has been shown to contribute to delayed treatment, underreporting, and increased risk of severe outcomes during peak transmission periods, thereby influencing observed fluctuations in morbidity and mortality (14).

The strong geographic disparities in WASH access explain much of the disease burden. Iganga District, with lower water access (53 percent) and the least functional handwashing facilities, also experiences the highest frequency of illness and delayed health-seeking. These conditions are consistent with global evidence showing that lack of access to safe water, sanitation, and hygiene correlates strongly with diarrhea, cholera, and typhoid risk (2, 3). Within this context, the WASH program sets a realistic quantitative target of a 2 percent increase in safe water access and an internal ambition of increasing toilet utilization by 10 percentage points by 2026, from 40 percent to 50 percent. The weakness is not only infrastructural, behavioral and economic barriers magnify risk. It was crystal clear that most respondents frequently reserve soap for washing clothes or bathing, suggesting that even where knowledge exists, competing priorities and poverty drive unsafe behavior. Evidence from similar WASH interventions suggests that such improvements are achievable within 2 years when practical barriers are addressed through subsidies, Community-Led Total Sanitation (CLTS), Market-Based Sanitation (MBS) approaches, and school WASH club engagement.

Health facilities illustrate the same contradiction. where 52 percent have functional handwashing stations, yet only 46 percent consistently have soap and water. Health workers acknowledged that hygiene enforcement collapses when supplies fluctuate. This aligns with regional studies showing that interruptions in water supply and soap availability reduce adherence to infection prevention and increase facility-acquired infections (13). Without reliable commodities, health promotion messaging collapses into empty advice.

Busoga compares poorly against national benchmarks. National household handwashing rates with soap and water increased from 29% in 2020 to 44% in 2024, while Busoga reached only 44% at baseline, 4–5 percentage points below national averages. Similarly, sanitation utilization (61.3% regional average) obscures deep inequities; Iganga (75%) and Jinja District (81.3%) show apparently strong latrine coverage, yet the quality and usability of facilities remain weak. School latrine-to-pupil ratios are 1:135 in Iganga and 1:83 in Jinja City, far exceed recommended standards of 1:40, undermining menstrual health and dignity. Literature from Kenya and Tanzania shows that overcrowded sanitation facilities increase absenteeism among girls and promote intermittent open defecation (15), mirroring the voices captured during FGDs.

Facility utilization also follows national and regional patterns: lower utilization is not only driven by distance, but by mistrust, stockouts, and poor cleanliness. Caregivers avoiding formal facilities in favor of herbs or private drug shops reflect common East African health-seeking behaviors, particularly where public facilities are understaffed or under-resourced (14).

School-level WASH conditions further amplify the observed disease burden and highlight critical institutional gaps that extend beyond households and health facilities. Evidence shows that many schools lack comprehensive WASH plans, adequate budgeting for operation and maintenance, and inclusive facilities such as gender-responsive sanitation and menstrual hygiene management infrastructure (12, 25). Poor functionality of handwashing facilities, inadequate safe water access points, and limited integration of WASH into school development planning have been consistently documented as key barriers to sustained hygiene behavior change (5, 25). These challenges are reflected in the present study, where extremely high latrine-to-pupil ratios and inadequate sanitation infrastructure undermine not only infection prevention but also school attendance, particularly among girls. Literature shows that inadequate and overcrowded sanitation facilities contribute to poor menstrual hygiene management and increased absenteeism among adolescent girls (13, 15). Schools, which should serve as centers for behavior change and health promotion, risk reinforcing poor hygiene practices when WASH systems are inadequate or poorly maintained. Integrating structured WASH planning, budgeting, and school WASH club engagement into education systems has been shown to improve both health and educational outcomes, positioning schools as a critical platform for breaking the cycle of waterborne disease transmission in communities (2, 12).

## Conclusion

The baseline and early midline data demonstrate that waterborne diseases in Iganga and Jinja remain a significant public health threat driven by structural deficiencies, behavioral gaps, and fragile WASH systems. While the average incidence for Busoga is 4.0%, the project areas exceed this level, with Jinja City at 5.4% and Iganga at 4.6%. This is backed up with the household survey which ranges from 3.8 to 5.9%. Diarrhea remains the most prevalent waterborne illness, followed by dysentery and typhoid, with cholera appearing episodically, especially during periods of flooding and water disruption. Household surveys reveal a hidden burden: for example, use of unimproved water sources, no treatment of, use of shared sanitation or open, lack of a handwashing facility and finally education at secondary level being protective, highlighting an underreported morbidity gap.

Despite moderate knowledge levels, 87% can name two diseases and 76% can list two preventive actions, behavior change remains inconsistent. Barriers include economic constraints, cultural normalization of diarrhea, poor school WASH infrastructure, and weak facility hygiene. The persistent underperformance of Iganga District across multiple indicators (water access, sanitation use, handwashing, facility provisioning) reinforces the association between WASH inequities and disease burden. These findings affirm that hardware alone is insufficient; sustained access, maintenance, reliable supply, and user-centered design are critical for impact.

Early observations show positive momentum; improved stakeholder ownership, the installation of WASH hardware, increased outpatient attendance, and strong satisfaction with capacity-building activities. However, the translation of awareness into consistent practice remains uneven. The project's goal of reducing waterborne disease incidence to 4.8% or below across the project areas, and increasing handwashing practice by 5%−10% across households, schools and health facilities, is realistic and attainable provided that implementation is aligned with the lived realities reflected in the baseline indicators.

The summary table demonstrates that Iganga District and Jinja City require differentiated targets, with Iganga needing deeper investment in safe-water access, sanitation reliability and consistent soap availability, while Jinja City requires sustained behavioral reinforcement and service management to maintain already higher coverage levels. Achieving the end-line targets will therefore depend on intensifying interventions in high-burden hotspots, ensuring continuous functionality of installed WASH infrastructure, and strengthening systems that enable daily behavioral practice, rather than relying on episodic sensitization campaigns.

## Recommendations

1) Prioritize functionality, maintenance, and continuity of WASH services, not infrastructure presence alone. Institutionalize routine maintenance, reliable supply chains, and resilient low-cost technologies to prevent reversion to unsafe practices during disruptions.
2) Shift from general awareness to habit formation using practical demonstrations, repeated reinforcement, and context-specific messaging. Engage influential community actors to challenge norms that normalize diarrhea and delay preventive action.
3) Ensure schools and health facilities consistently reinforce hygiene behaviors through adequate sanitation infrastructure, reliable supplies, and clear accountability. Strengthen student-led WASH platforms and IPC supervision to normalize and sustain healthy practices.
4) Formalize collaboration with local governments, health and education sectors, and community structures to clarify roles and ensure long-term maintenance. Leverage existing platforms such as CLTS, VHTs, and school health clubs to support scale-up and ownership.
5) Integrate routine surveillance with household and qualitative data to detect underreported burden and seasonal risks early. Institutionalize community feedback mechanisms to support adaptive programming and accountability.

## Data Availability

The raw data supporting the conclusions of this article will be made available by the authors, without undue reservation.
